# Fusiform Activity Distinguishes Between Subjects With Low and High Xenophobic Attitudes Toward Refugees

**DOI:** 10.3389/fnbeh.2020.00098

**Published:** 2020-09-11

**Authors:** Ladislav Kesner, Iveta Fajnerová, Petr Adámek, Martin Buchtík, Dominika Grygarová, Jaroslav Hlinka, Pavel Kozelka, Tereza Nekovářová, Filip Španiel, Jaroslav Tintěra, Aneta Alexová, David Greguš, Jiří Horáček

**Affiliations:** ^1^Applied Neurosciences and Neuroimaging, National Institute of Mental Health, Klecany, Czechia; ^2^Department of Art History, Faculty of Arts, Masaryk University, Brno, Czechia; ^3^Third Faculty of Medicine, Charles University, Prague, Czechia; ^4^Institute of Sociology, Czech Academy of Sciences, Prague, Czechia; ^5^Institute of Computer Science of the Czech Academy of Sciences, Prague, Czechia

**Keywords:** refugee crisis, xenophobia, attitude, conscientiousness, fMRI

## Abstract

This study analyzes how people's attitudes to the European refugee crisis (ERC) correspond to selected psychological state and trait measures and impact the neural processing of media images of refugees. From a large pool of respondents, who filled in an online xenophobia questionnaire, we selected two groups (total *N* = 38) with the same socio-demographic background, but with opposite attitudes toward refugees. We found that a negative attitude toward refugees (high xenophobia - HX) was associated with a significantly higher conscientiousness score and with a higher trait aggression and hostility, but there was no group effect connected with empathy, fear, and anxiety measures. At the neural level we found that brain activity during the presentation of ERC stimuli is affected by xenophobic attitudes—with more xenophobic subjects exhibiting a higher BOLD response in the left fusiform gyrus. However, while the fMRI results demonstrate increased attention and vigilance toward ERC-related stimuli in the HX group, they do not show differentiated patterns of brain activity associated with perception of dehumanized outgroup.

## Introduction

Migration is a critical global challenge with vast social, political, and economic implications. This was dramatically highlighted by the recent massive influx into Europe of refugees from Syria, Afghanistan, and other near-eastern countries, which in 2015 escalated into what the media dubbed the “European refugee crisis” (hereinafter ERC). While negative and hostile attitudes toward immigrants are common (Stephan et al., 1999) and widespread anti-immigrant attitudes in Europe predate the ERC (McLaren, 2003; Meuleman et al., 2009; Fasel et al., 2013), this recent wave of migration has been accompanied by a dramatic surge in negative feelings toward refugees across many European countries. In terms of intergroup threat theories (Blumer, 1958; Riek et al., 2006), immigrants have often been perceived as a threatening out-group—representing a threat that is both potentially real (“a source of terrorism”) and symbolic (“a challenge to identity and culture”).

Among the most critical aspects of migration is the impact of media representations on the formation and maintenance of attitudes and specifically their role in activating and spreading xenophobia, hostility, and a crisis mentality (Gale, 2004; Gross, 2008; Boomgaarden and Vliegenthart, 2009; Bleiker et al., 2013; Esses et al., 2013; Rane et al., 2014; Bodas et al., 2015; Elbaz and Bar-Tal, 2016). One of the defining features of the ERC has been the extensive and deliberate manipulation of people's attitudes and feelings toward refugees, often in order to promote particular political interests. Because of the massive media coverage (and in some countries the arrival of large numbers of refugees during ERC), they have come to represent a highly salient social group. However, the ERC provides a unique opportunity to study the effect of media representations on attitude formation, as it differs in some significant ways from the type of in-group/out-group scenarios typically examined in previous research and this is particularly true in the specific context of our study.

A key effect of the political messages and media representations that surfaced during the ERC was that the ethnically and religiously diverse populations of refugees were transformed into the homogenized image of a “Muslim” or “Arab” immigrant. Islam and Muslims are typically presented and perceived as a threat to national identity, culture, and security (Velasco González et al., 2008). Several studies have examined this bias toward the Muslim outgroup, establishing, for instance, that subjects were quicker in deciding to shoot an ambiguous armed target if primed with the category Arab or Muslim (vs. no category priming) or if the target was wearing Islamic head dress (Dalton et al., 2005; Unkelbach et al., 2008; Dominguez et al., 2017). On the other hand, the perception of ERC refugees does not involve direct racial bias or hostilities between deeply encoded traditional conflict groups (such as Arabs vs. Israelis), which have been the subject of extensive research (Xu et al., 2009; Bruneau and Saxe, 2010; Mathur et al., 2010; Contreras-Huerta et al., 2013; Fox et al., 2013; Zuo and Han, 2013) and this is particularly relevant in the specific local context of our study. Unlike the European countries that have a large immigrant population, the Czech Republic has only a tiny Arab and Muslim minority and there is no history of conflict between that minority and the native population. No more than several dozen refugees have come to the country since the start of the crisis, all of them concentrated in asylum centers. The widespread display of negative attitudes toward refugees (among the most hostile in the European Union) (cf. Eurobarometer, 2015; Bruneau et al., 2017) that occurred in the public space and on social media during the crisis therefore cannot be a reflection of personal experience and can only be attributed to the effect of media representations and in particular to the way political actors and the media elicited this hostility. Public opinion on the ERC has also been polarized, with the minority view expressing positive attitudes toward refugees. The questions we need to ask then are (i) how attitudes toward refugees coming to Europe (attitudes induced almost exclusively by media representations) modulate brain response to representations of the ERC and (ii) whether this response exhibits patterns of neural activity characteristic of in/out group biases, which have been described in previous social psychology and intergroup neuroscience research (e.g., Eberhardt et al., 2004; Amodio, 2008, 2014; Stanley et al., 2008; Bruneau et al., 2012; Falk and Lieberman, 2013; Molenberghs, 2013; Cikara and Van Bavel, 2014; Jost et al., 2014; Halperin and Sharvit, 2015).

To address these issues, this study examined how *a priori* attitudes toward refugees modulate the perception of affective stimuli depicting the ERC and how this modulation is manifested in the objective correlates of brain activity. First, out of a large pool of respondents we selected two groups at opposite ends of the scale of xenophobia toward refugees. To compare high-level and low-level xenophobia subjects for psychological domains that potentially mediate xenophobia, all of the participants were evaluated for empathy, hostility, anxiety, and fear, as well as for personality traits. In the fMRI part of this study, we examined how the BOLD response to media images relating to the ERC was modulated by explicitly acknowledged attitudes toward refugees. The participants looked at four categories of real media images (160 in total) obtained from various mainstream media outlets as stimuli, and two categories were recognizably connected with the ERC. Participants were instructed to internalize and feel the emotion evoked by the stimuli. Data acquisition was carried out in the spring 2016, at a time when the salience of the ERC in the public space was strongest.

For the neuroimaging component of the research, we predicted top-down modulation of neural processing by attitudes toward refugees in two sets of images depicting the ERC. First, we expected the threat-related response to vary depending on the content of the images. A number of earlier studies established that there is a neural response to threatening (typically racial) out-groups and that it occurs particularly in the amygdala (Chekroud et al., 2014; Bruneau, 2015 for review), and based on these findings we expected that images depicting masses of refugees would be perceived as inherently threatening and in the group of more xenophobic participants/subjects would elicit brain activation in the amygdala and possibly also the insula.

Second, regarding the response to images showing close-ups of emotional expressions, our aim was to test two alternative (but mutually compatible) possibilities of how a person's attitude toward refugees modulates brain response. One possibility was that in the less xenophobic group the response to refugees in pain and distress will exhibit the well-documented pattern of ingroup empathy bias (Xu et al., 2009; Cheon et al., 2011; Cikara and Fiske, 2011; Gutsell and Inzlicht, 2012; Molenberghs, 2013; Cikara and Van Bavel, 2014; Han, 2018; Molenberghs and Louis, 2018) registering in enhanced activity in the areas of the brain associated with affective empathy (particularly anterior insula and anterior cingulate cortex) and cognitive empathy (medial prefrontal cortex, temporoparietal junction) in subjects from this group. We were particularly interested in the role of the fusiform gyrus, which responds selectively to face stimuli based on their group membership. Ample evidence exists that processing in the fusiform gyrus is transiently altered by top-down cognitive biases and motivational influences, including stereotypes and attitudes (e.g., Bruneau et al., 2012; Brosch et al., 2013; Stolier and Freeman, 2016). There are several studies that have produced evidence of own group bias, whereby in-group faces are more strongly encoded and better remembered than those of out-group members (Young and Hugenberg, 2010; Hugenberg et al., 2013; Ratner and Amodio, 2013; Kawakami et al., 2017). Under this scenario, and in line with previous research that has found increased activation in the FG in response to racial (Golby et al., 2001; Cunningham et al., 2004) or otherwise defined in-group compared to out-group faces (Van Bavel et al., 2008, 2011), we posited that in-group bias pattern in the low xenophobia group would be manifested as enhanced activation of the FG, suggesting a more in-depth processing and greater individuation of refugees. Higher measures of state/trait empathy in LX group could be expected to correlate with the imaging data.

The other possibility was that the pattern of brain response to the emotional faces of refugees would yield more robust results in the high xenophobic group, an expectation borne out of the well-established fact that there are pervasive biases in visual attention to highly salient and stereotype-congruent stimuli (Brosch and Sharma, 2005; Maner et al., 2005; Ackerman et al., 2006; Blanchette, 2006). Several psychological studies have produced converging evidence that out-group members who are perceived negatively thereby elicit increased attention, vigilance, and avoidance (Eberhardt et al., 2004; Donders et al., 2008; Senholzi et al., 2015; Chang et al., 2016). Under this scenario, it could be expected that this increased attention and processing would in the more xenophobic group register in the fusiform gyrus but not in the amygdala, as we reasoned that emotional faces would not be perceived as inherently threatening by our subjects. Third, we predicted that in two categories of stimuli showing images not related to the ERC (i.e., threatening images of Islamic terrorists and pain and suffering in other contexts), the empathic and threat-related response would not correlate with explicit attitudes, but rather with the individual state and traits measures of fear and anxiety (in the case of threatening images of ISIS) and empathy (in the case of images of non-ERC victims).

## Materials and Methods

### Sample Recruitment

**Figure 1:**
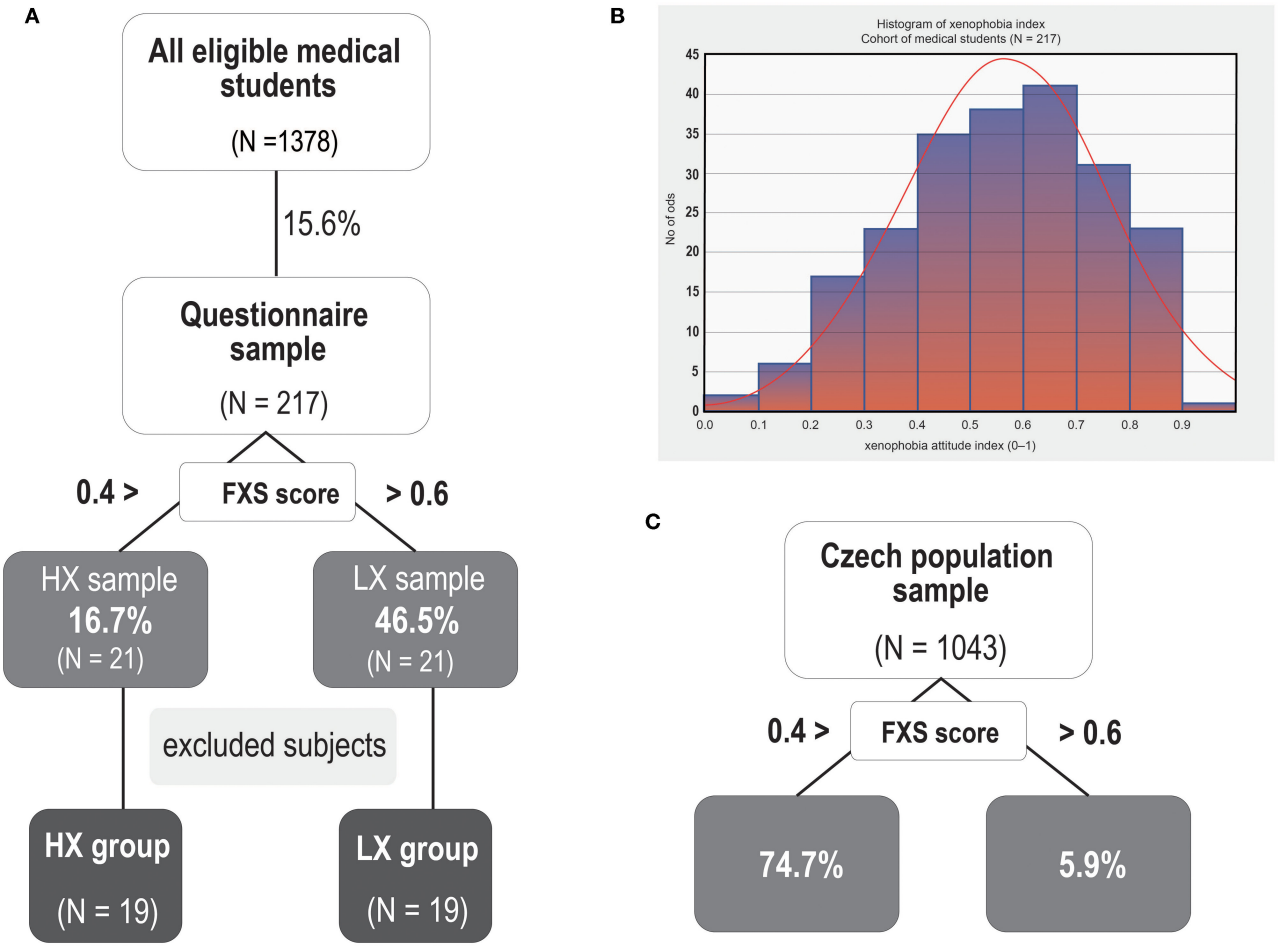
Profile of the experimental sample. **(A)** Experimental sample distribution and illustration of the recruitment process. **(B)** Histogram depicting the distribution of results for the Fear-Based Xenophobia (FXS) Scale (a total score ranging from 0 to 1) in the sample of 217 subjects who filled in the short online questionnaire. **(C)** Distribution of the two opposite poles of FXS scores in the selected sample of the Czech population. Note that when compared to a representative sample of the Czech general population, our participants showed substantially lower levels of xenophobia [with both a lower rate of high xenophobia scores (below 0.4) and a higher rate of the least xenophobic scores (0.6–1) (see % values in diagram **A,B**)]. Note that the threshold values are different from the stricter inclusion criteria that were applied in the recruitment process (below 0.25 or above 0.75).

We chose Czech medical students as a target group in order to ensure the sample was homogeneous in terms of age, education, and social status. We used a two-step procedure to recruit the participants. First, we contacted all students attending the First Faculty and Third Faculty of Medicine at Charles University in Prague through the school email. We asked them to participate in the study and fill in a short online questionnaire designed to measure the participant's level of xenophobia and opinions on the ERC. To assess attitudes toward immigrants we used the Fear-based Xenophobia Scale—FXS (Van der Veer et al., 2011, 2013), which is probably the only measure that is used internationally and was developed and validated for psychometric testing (Canetti-Nisim and Pedhazur, 2003; Van der Veer et al., 2011—Czech translation Tabery et al., 2015) and that has also been shown to be reliable and valid for use on the Czech population (Kunc, 2016). It is a 9-item scale covering different aspects of xenophobia (i.e., political fear, personal fear, fear of cultural change, losing identity, and disloyalty) measured on a 7-point Likert-type scale. The xenophobia index was computed as the average of valid items and then transformed to a <0; 1> scale, where 0 represents the most xenophobic attitudes and 1 the least xenophobic attitudes. The FXS scale measures only fear-related emotional reactions to foreigners (Van der Veer et al., 2011), while other dimensions, such as hate or contempt, are not taken into account (Van der Veer et al., 2013). The questionnaire was completed by 217 students (the response rate was 15.6%). In order to examine the two opposite extremes of xenophobic attitudes to the ERC, we invited the 21 respondents with the highest xenophobic score [the high-xenophobia (HX) group scored below 0.25] and the 21 respondents with the lowest xenophobic score [the low-xenophobia (LX) group scored above 0.75] to participate in the study (see Figure 1 for details on the participants and defining HX and LX) (the threshold values were determined according to the distribution of the xenophobia score in the original group of 217 students).

### Participants

Forty-two adults (24 women, 18 men), ranging in age from 19 to 26 (mean age = 22.3 years), took part in the study. All the participants had normal or corrected to normal vision, and all were screened to exclude people with any contraindications for MRI scanning or with any history of neurological trauma or psychiatric disorder. No subject reported taking any medication that affects the central nervous system. Informed consent was approved by the Ethical Committee of the National Institute of Mental Health and obtained from all the participants. Four of the tested subjects were excluded from the final analysis on the grounds of a clinically positive MRI report or poor-quality MRI data. The statistical analysis was performed on group size *N* = 38 (18 males and 20 females; HX: 10 males and 9 females, LX: 8 males and 11 females).

### Psychometric Methods

Before undergoing the fMRI scanning, participants completed a battery of online questionnaires using the custom-made online Forms app (https://forms.nudz.cz/). The psychometric measures included: (1) the 16-item Toronto Empathy Questionnaire (TEQ) assessing empathy as a primarily emotional process (Spreng et al., 2009), which was translated into Czech from the original and then back-translated into English in order to ensure the accuracy of the translation; (2) a Czech version (Němcová, 2008) of the Buss-Perry Aggression Questionnaire (BPAQ) measuring aggression and hostility (Buss and Perry, 1992) based on 29 items subgrouped into four factors (Physical aggression/PA, Verbal aggression/VA, Anger/A, and Hostility/H); (3) the short 21-item Fear Questionnaire (FQ) was used to measure the main types of phobia (Marks and Mathews, 1979); (4) the State-Trait Anxiety Inventory (STAI) was administered, which is an introspective psychological inventory consisting of 40 self-report items aimed at state-trait/S-T distinction of anxiety affect (Müllner et al., 1980); (5) the 60-item Neo Five Factor Inventory (NEO-FFI) assessing the personality traits of extraversion, agreeableness, conscientiousness, neuroticism, and openness to experience (Goldberg, 1993; Hrebíčková and Urbánek, 2001).

### The fMRI Experiment

#### Stimuli Preparation

**Figure 2:**
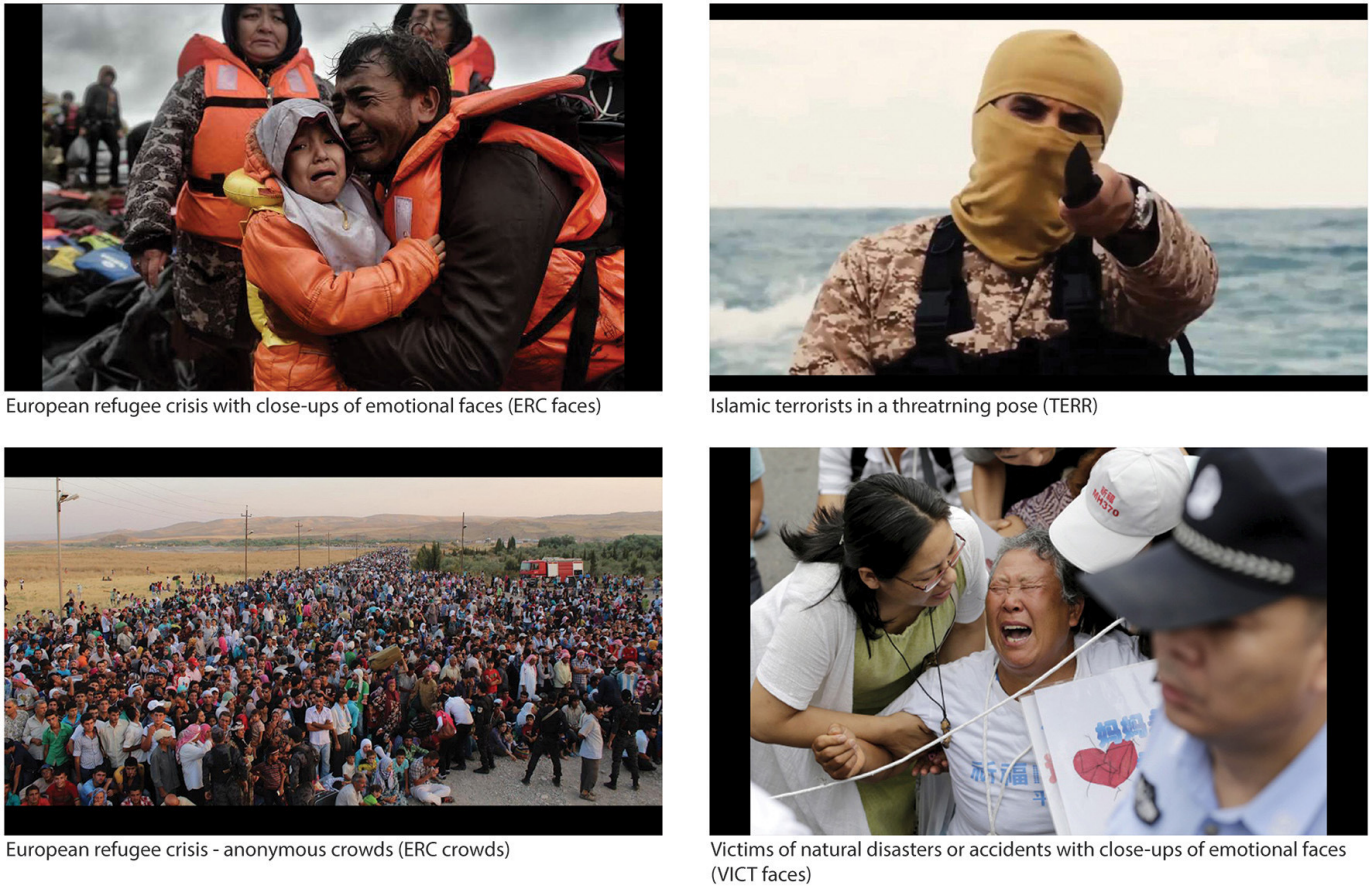
Schema of sample stimuli. (i) Photographs of refugees with close-ups of their emotional faces (ERC FACES); (ii) photographs depicting anonymous crowds of refugees (ERC crowds); (iii) photographs of Islamic terrorists in threatening and violent poses (TERR); and (iv) photographs of victims of another conflict, disaster, or accident with close-ups of their emotional faces (VICT FACES).

The participants were exposed to 160 documentary high-resolution color photographs obtained from various mainstream media outlets as stimuli: 40 photographs of the ERC with close-ups of emotional faces (ERC FACES); 40 photographs of the ERC showing an anonymous crowd (ERC CROWDS); 40 photographs of Islamic terrorists in a threatening pose (TERR); 40 photographs of victims of natural disasters or accidents with close-ups of emotional faces (VICT FACES); 80 non-artistic photographs of various landscapes were used as a control condition (Figure 2). The aspect ratio of the photographs was converted on the longest side to 1,024 pix and 300 dpi using a bicubic sharper algorithm and Adobe Photoshop CS5. The photographs were chosen on the basis of a color and brightness analysis performed by our own Matlab color-brightness algorithm in order to eliminate undesirable brain activation in response to glare or substantial color variation (Tsubomi et al., 2012). In order to enable direct comparison, the two sets of ERC FACES and VICT FACES photographs were matched by the authors for the pictorial content in following parameters: the presence of facial details, number of depicted persons, valence, and intensity of emotional facial expression, type of catastrophe captured, and specific scenes if possible (e.g., emotionally expressive people being helped, desperate people in the water, people wrapped up in blankets etc.), and general composition.

**Figure 3:**
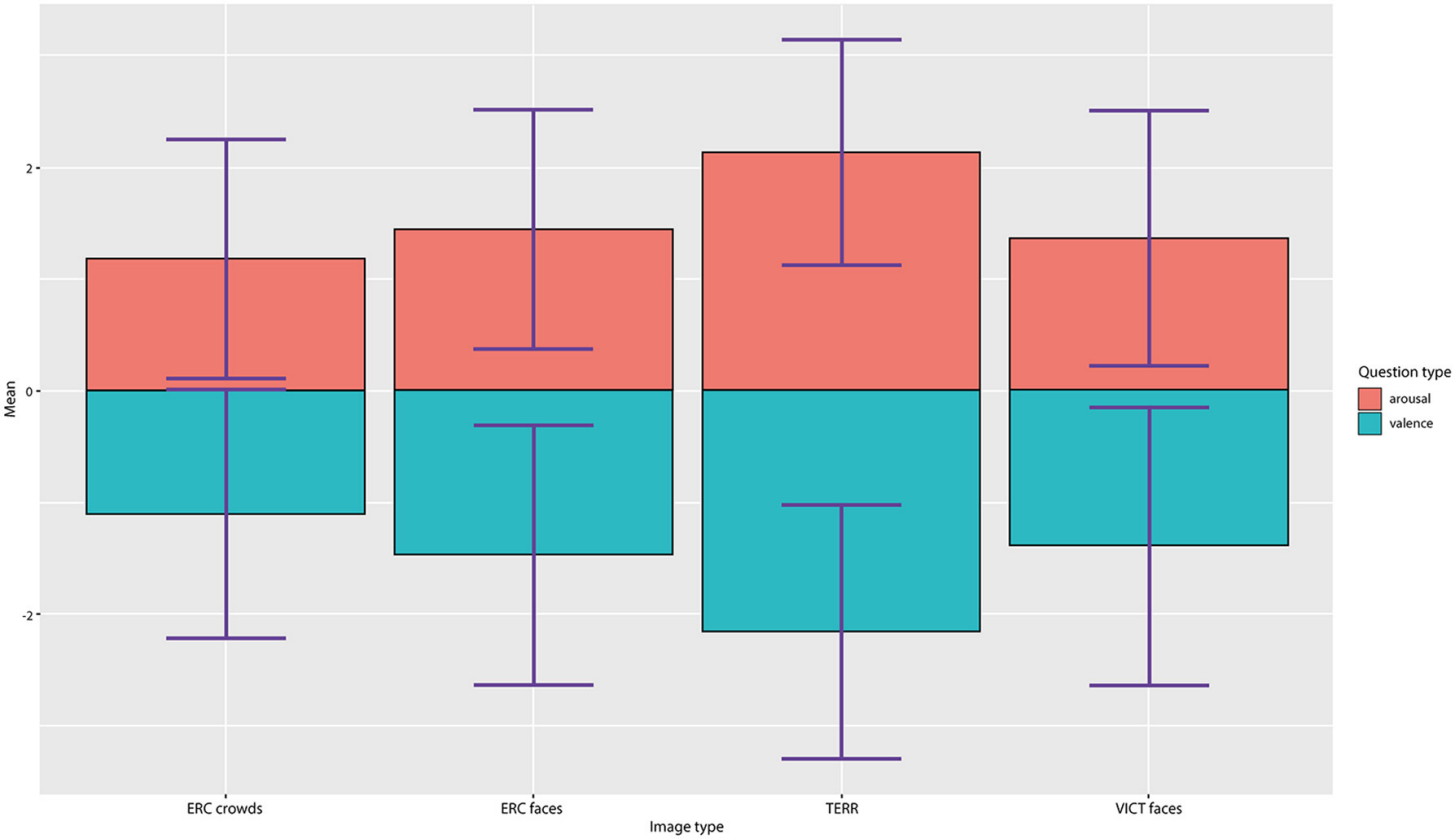
Valence and arousal ratings. Results of the ratings of valence and arousal by a 7-point Likert scale (ranging from −3 to +3) evaluated for all four categories of photographs.

**Figure 4:**
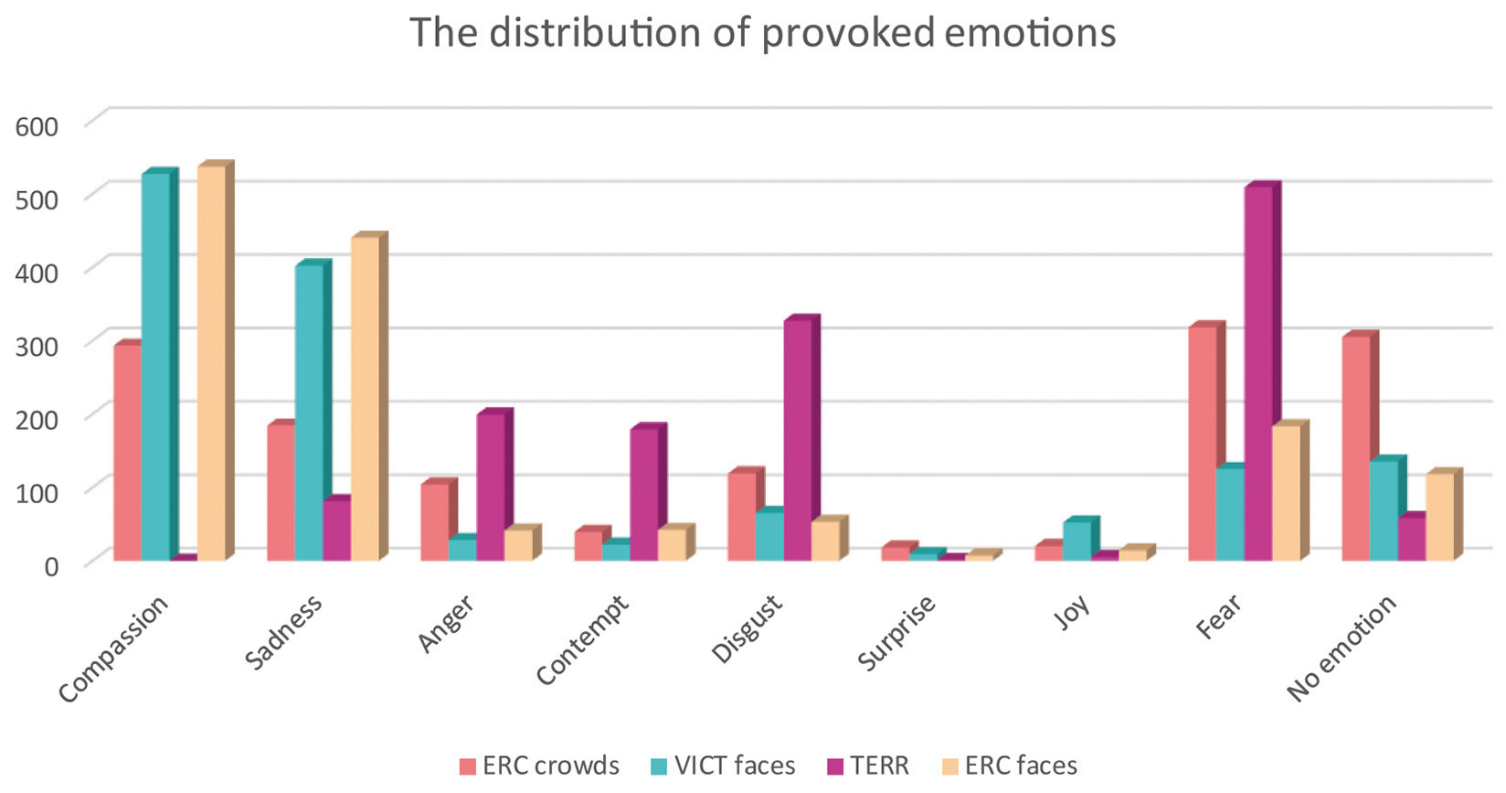
The distribution of emotions provoked by the four categories of photographs. Individual categories of photographs are presented in various colors. The figure shows cumulative number of responses of the specific emotion in each category of photographs (*N* = 1,400 responses in total for each category).

The final set of experimental stimuli was assessed by 35 independent raters (mean age = 24.4, *sd* = 6.23) to evaluate inter-subject concordance. Each image was evaluated for emotional valence (ranging from very unpleasant to very pleasant) and arousal (very calm/very aroused) on a 7-point Likert scale (−3 to +3). Kendall's coefficient of concordance showed (a) good degree of agreement for all the image categories (*W* = 0.683, *p* < 0.001). In the evaluations, the TERR photographs had the strongest arousal effect (mean arousal = 2.08) and the least pleasant (mean valence = −2.06), whereas the photographs of anonymous ERC CROWDS elicited the most neutral response out of all of the categories (mean arousal: 1.08, mean valence: −1.02) (see Figure 3—Valence and arousal ratings). The difference between the ERC FACES and the VICT FACES photographs assessed by raters was negligible and insignificant both in the valence dimension [VICT FACES: mean = −1.387; ERC FACES: mean = −1.462 (*t* = 1.530, *df* = 136, *p* = 0.126)] and in the arousal dimension [VICT FACES: mean = 1.370; ERC FACES: mean = 1.448 (*t* = −1.853, *df* = 136, *p* = 0.064)]. Therefore, we considered VICT FACES and ERC FACES balanced in terms of their emotional impact. The participants also selected a specific emotion (in a single-choice question: compassion/empathy, sadness, anger, contempt, disgust, surprise, joy, fear, or no emotion present) and how strong (on a scale of 1–7) a sense of this emotion was elicited by each photograph (see Figure 4—Distribution of provoked emotions; note that the intensity ratings are not reported). The most frequent answers in response to the TERR photographs were fear, anger, contempt and disgust, while compassion/empathy and sadness were the most frequent answers in response to both the VICT FACES and the ERC FACES photographs. The ERC CROWDS photographs elicited mostly fear, compassion, or no emotion at all.

#### Procedure and Image Acquisition

We used a block design paradigm, and in each block, four different photographs of each condition (ERC CROWDS, ERC FACES, VICT FACES, and TERR) were shown, with two landscape images as a control condition in between. Altogether ten blocks of stimuli were presented for each condition. Each stimulus was presented for 6 s, once in the course of the experiment in pseudo-randomized order. Participants were instructed to consciously acknowledge and feel the emotion evoked by the stimuli. Right after the fMRI imaging was performed, each participant was asked to class the stimuli into three groups (ERC, TERR, and VICT FACES) to make sure that his/her recognition of the stimuli content corresponded to what the photographs actually depicted.

Structural and functional data were collected on a 3T Siemens Prisma scanner (Siemens Medical Systems, Erlangen, Germany) equipped with a 64-channel head coil. The stimuli were rear-projected onto a mirror mounted on the head coil. Functional images (BOLD) were acquired using a gradient-echo echo-planar imaging sequence (GE-EPI) covering the whole brain with 46 slices and a voxel size of 3 × 3 × 3 mm^3^. This functional sequence had the following parameters: FOV = 192 mm, TR/TE = 3,000/30 ms, a flip angle of 80°, a bandwidth of 2,520 Hz/pixel, an iPAT acceleration factor of 2. During the functional measurement total of 480 scans of brain volumes were acquired, which resulted in 24 min of acquisition time. Whole-brain anatomical scans were also acquired using a 3D T1-weighted magnetization-prepared gradient echo sequence (MP-RAGE), consisting of 240 sagittal slices with a resolution of 0.7 × 0.7 × 0.7 mm^3^ (TR/TE/TI = 2,400/2.34/1,000 ms, FOV = 224 mm, total acquisition time 7:40 min), and a 3D T2-weighted SPACE sequence with the same resolution (TR/TE = 3,200/564 ms; total acquisition time 7:55 min).

#### The fMRI Data Analysis

The fMRI data were analyzed first using version 12 of Statistical Parametric Mapping software (SPM12; http://www.fil.ion.ucl.ac.uk/spm/software/spm12/) implemented in version R2016b MATLAB (MathWorks). The statistical analysis of the data on all the participants was conducted by entering each participant's pre-processed functional data, which were realigned, normalized and finally spatially smoothed using a 6 × 6 × 6 mm Gaussian kernel into a generalized linear model. Low-frequency noise was removed using a high-pass filter (128 s). The first-level design matrix contained factors that model the hemodynamic response function for ERC CROWDS, ERC FACES, TERR, and VICT FACES conditions contrasted against landscape blocks. The contrast images (linear combinations of β images) were then subjected to second-level analyses to determine the condition-specific regional responses at the group level. We used a full-factorial model that included group (2 levels: HX and LX) and condition (4 levels: ERC CROWDS, ERC FACES, TERR, and VICT FACES) factors. We set the level of significance at *p* ≤ 0.05 family-wise error (FWE), corrected for all gray matter (GM) voxels defined by the study-specific mean segmented GM volume.

Functional connectivity was analyzed using a seed-driven approach that employed CONN version 15.h connectivity software (www.nitrc.org/projects/conn/). Data were pre-processed according to the same parameters that were applied to SPM12. Physiological and other spurious sources of noise (a signal from a region in the cerebrospinal fluid, white matter, and the whole brain signal) were estimated using the (implemented) component-based method and were removed together with the movement-related covariates (Whitfield-Gabrieli and Nieto-Castanon, 2012). The residual BOLD time series were band-pass filtered over a low-frequency window of interest (0.008–0.09 Hz). The correlation maps produced by computing the Pearson correlation coefficients between the residual BOLD time course from the functionally defined specific seed of the left fusiform gyrus cluster (resulting from the SPM analysis, see below), and all other gray matter voxels were converted to normally distributed *Z* scores using Fisher transformation. The *Z* maps were submitted to a random-effects full factorial model to address the interaction between the groups and conditions using an FWE-corrected threshold at cluster level *p* ≤ 0.05.

#### Statistical Analyses

The Student's *t*-test was used to analyze group differences in age and in all psychometric variables. The significance level was set at *p* < 0.05. First, the total scores for the individual scales were calculated in order to test our main hypothesis. For additional analysis, the *t*-test was used on individual subscales. In this case, the Bonferroni correction was used to control the effect of repeated measures.

## Results

### Demographic and Psychometric Data

The participants recruited for the LX and the HX groups were matched for sex and age wherever possible based on the demographic distribution of the available study sample.

The *per protocol* selection of participants at opposite ends of the FXS distribution in the original sample of 217 medical students incidentally led to slight age differences between the two groups. We discuss this fact in the limitations section. The groups show a comparable distribution of males and females in the LX and the HX groups according to Cramer's *V*-test (for more details, see Table 1). It should be noted that there was no difference in the FXS scores between males and females (*t* = −0.49, *p* = 0.63).

**Table 1:** A comparison of demographic and psychometric data for the LX and HX groups.

**Variable**		**Group Mean (SD)**		***t*-test or Cramer's *V*-value**	***p***
		**HX group**	**LX group**		
***N***		19	19		
**Sex**	**Females**	9	11	0.41^+^	0.52
	**Males**	10	8		
**Age:** Mean (SD) min–max		23.0 (1.86) 19–26	21.37 (1.46) 19–24	−3.01	0.005^**^
**FXS score**		0.23 (0.09)	0.84 (0.04)	4.37	0.003^**^
**Method**	**Psychometric variable**	**HX**	**LX**	***t***	***p***
**TEQ**	**TPS**	**45.21 (5.42)**	**48.37 (5.76)**	**−1.74**	**0.091**
**BPAQ**	**TPS**	**43.00 (13.61)**	**32.21 (11.25)**	**2.66**	0.011*
	PA	10.00 (4.56)	6.32 (4.49)	2.51	0.017*
	VA	11.21 (3.60)	9.21 (3.19)	1.81	0.078
	A	10.21 (3.98)	9.84 (5.04)	0.25	0.804
	H	11.58 (5.73)	6.84 (4.32)	2.88	0.007*
**STAI**	S	36.37 (7.62)	38.16 (9.43)	−0.64	0.524
	T	37.89 (7.25)	38.16 (7.97)	−0.11	0.916
**FQ**	**TPS**	**49.05 (20.67)**	**39.95 (17.48)**	**1.47**	**0.151**
	AD	17.58 (9.58)	16.63 (9.67)	0.30	0.763
	SC	12.53 (6.41)	11.47 (5.76)	0.53	0.598
	BI	9.53 (5.39)	6.47 (4.56)	1.88	0.068
	A	9.42 (8.30)	5.37 (4.45)	1.88	0.069
**NEO-FFI**	N	−0.22 (0.89)	−0.35 (1.20)	0.42	0.675
	E	0.10 (1.12)	0.05 (0.99)	0.13	0.898
	O	0.35 (0.88)	0.80 (1.09)	−1.49	0.145
	A	0.03 (1.11)	0.75 (0.97)	−2.20	0.034
	C	1.02 (0.85)	−0.07 (1.27)	3.18	0.003*

Total profile score (TPS) values for individual psychometric methods are presented in bold. All significant results for individual psychometric measures surviving the Bonferroni correction for multiple comparisons are marked by asterisk (*p < 0.05, **p < 0.01); ^+^, Cramer's V value.

*Abbreviations: FXS, Fear-based Xenophobia Scale; TEQ, Toronto Empathy Questionnaire; BPAQ, Buss-Perry Aggression Questionnaire; PA, physical aggression; VA, verbal aggression; A, anger; H, hostility; STAI, State-Trait Anxiety Inventory; S, state anxiety; T, trait anxiety; FQ, Fear Questionnaire; AD, anxiety-depression; SC, social phobia; BI, blood and injury phobia; A, agoraphobia; NEO-FFI, NEO Five-Factor Inventory; N, neuroticism; E, extraversion; O, openness to experience; A, agreeableness; C, conscientiousness*.

#### Psychological Differences Between the Groups With High (HX) and Low (LX) Xenophobia Levels

The HX group showed the following significant differences from the LX group: a higher score in the BPAQ aggression questionnaire (*p* < 0.05), in particular on the physical aggression (PA, *p* = 0.017) and hostility subscales (H, *p* = 0.007). We found no other significant group-specific differences in empathy (TEQ), anxiety level (STAI state and trait), or fear and phobia (FQ). Regarding the personality traits identified by NEO-FFI, we found a higher HX score for the Conscientiousness subscale (NEO-FFI-C: *U* = 109.0; *z* = −2.08; *p* = 0.038). For more detailed results, see Table 1.

### Neuroimaging Results

#### The Main Effects of the Content of the Documentary Photographs

**Figure 5:**
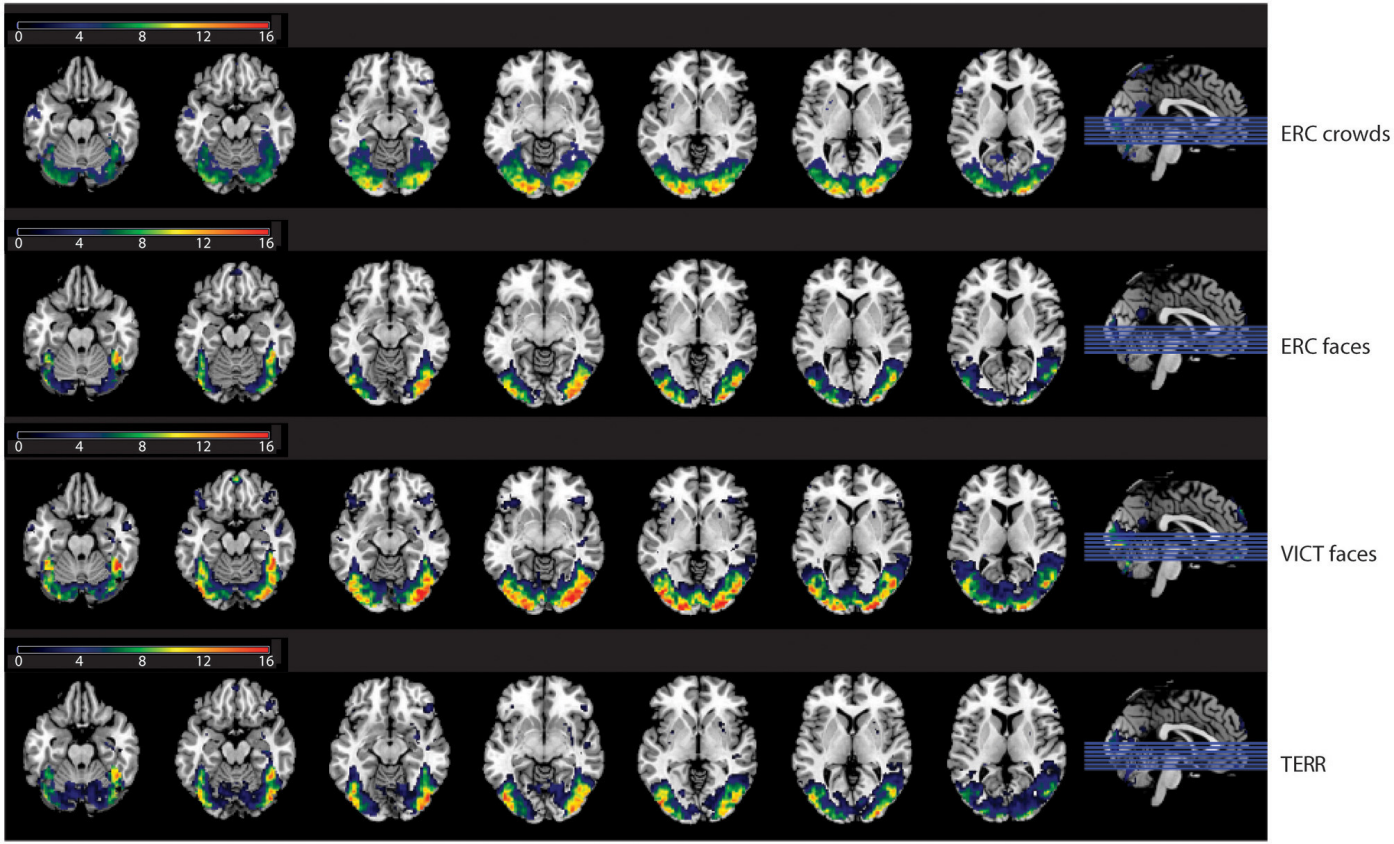
The effect of the content of the documentary photographs on brain activity. The results are mapped on an anatomical template (MNI *z* = −22, −17, −12, −7, −2, 3, 8, FWE *p* ≤ 0.05). The color bars on the left show *t*-values. ERC FACES, European refugee crisis with close-ups of emotional faces; ERC crowds, European refugee crisis showing an anonymous crowd; TERR, Islamic terrorists in a threatening pose; VICT FACES, victims of natural disasters or accidents with close-ups of emotional faces. The images were rendered using MRIcron software and ch2bet brain template (https://www.nitrc.org/projects/mricron/).

Compared to the control condition, the photographs of refugees with close-ups of emotional faces (ERC FACES) elicited brain activation in the bilateral visual occipital cortex, the precuneus, and the right middle temporal gyrus (FWE *p* ≤ 0.05). Images showing an anonymous crowd of refugees (ERC CROWDS) elicited a response bilaterally in the occipital visual cortex and the precuneus (FWE *p* ≤ 0.05). Photographs of Islamic terrorists in a threatening pose (TERR) activated the bilateral visual occipital cortex, the right inferior frontal gyrus, and the right amygdala (FWE *p* ≤ 0.05). Images of victims of natural disasters or accidents with close-ups of emotional faces (VICT FACES) elicited activation in the bilateral middle and the inferior occipital gyrus, the gyrus rectus, and the inferior frontal gyrus, the right middle temporal and the calcarine sulcus. On the left side, we identified activation in the precuneus (FWE *p* ≤ 0.05, Figure 5, Table 2).

**Table 2:** The effects of the content of the documentary photographs on brain activity.

	**Cluster vx**	**p (FWE)**	**T**	**Z**	***x***	***y***	***z***	**R or L**	**Brain region (local maximum)**
ERC crowds: activation	15281	0.000	16.30	Inf	−14	−94	−4	L	Calcarine, inferior, and middle occipital gyrus
	6	0.000	15.89	Inf	22	−98	8	R	Superior occipital gyrus
	10	0.000	14.63	Inf	−22	−100	2	L	Middle occipital gyrus
	471	0.000	6.40	6.00	2	−58	18	R	Precuneus
	369	0.005	5.51	5.24	−2	−60	16	L	Precuneus, calcarine
ERC faces: activation	5016	0.000	16.19	Inf	40	−86	−10	R	Inferior occipital and middle temporal gyrus
	5019	0.000	15.48	Inf	−48	−76	4	L	Middle occipital gyrus
	10	0.000	10.03	Inf	−24	−100	2	L	Middle occipital gyrus
	269	0.000	6.64	6.19	2	−60	24	R	Precuneus
	87	0.038	5.01	4.80	−2	−58	24	L	Precuneus
ERC faces: deactivation	979	0.000	6.27	5.89	6	32	26	R	Anterior cingulate gyrus
	461	0.000	6.21	5.84	4	−32	46	R + L	Middle cingulum
	1872	0.001	5.99	5.65	40	52	14	R	Middle frontal gyrus
	88	0.001	5.80	5.49	−28	−44	−12	L	Fusiform gyrus
	173	0.002	5.75	5.45	32	−42	−12	R	Fusiform gyrus
	824	0.003	5.68	5.39	−4	36	0	L	Anterior cingulate gyrus
	631	0.003	5.63	5.35	52	−38	56	R	Inferior parietal lobule
	1443	0.013	5.29	5.05	56	2	4	R	Superior temporal gyrus
	1225	0.024	5.13	4.90	−36	36	12	L	Inferior, middle, and superior frontal gyrus
	328	0.035	5.03	4.82	−38	−64	54	L	Inferior parietal lobule
	70	0.045	4.97	4.76	−58	−52	−16	L	Inferior temporal gyrus
	138	0.049	4.94	4.74	10	20	2	R	Caudate
TERR: activation	13433	0.000	15.04	Inf	40	−86	−10	R	Inferior occipital gyrus
	6	0.000	9.81	Inf	22	−98	8	R	Superior occipital gyrus
	10	0.000	8.90	Inf	−20	−96	4	L	Middle occipital gyrus
	144	0.004	5.58	5.30	46	32	−14	R	Inferior frontal gyrus
	88	0.009	5.37	5.12	30	−2	−12	R	Amygdala
TERR: deactivation	694	0.042	4.98	4.78	38	56	8	R	Middle frontal gyrus
VICT faces: activation	17880	0.000	16.96	Inf	−48	−74	4	L + R	Middle occipital and middle temporal gyrus
	6	0.000	12.82	Inf	22	−98	8	R	Superior occipital gyrus
	10	0.000	11.58	Inf	−20	−98	4	L	Middle occipital gyrus
	44	0.000	6.42	6.01	2	52	−20	R	Gyrus rectus
	803	0.000	6.11	5.75	50	24	22	R	Inferior frontal gyrus
	19	0.007	5.42	5.16	−2	54	−20	L	Gyrus rectus
	293	0.012	5.31	5.06	−38	24	−16	L	Inferior frontal gyrus
	84	0.024	5.13	4.91	−2	−58	22	L	Precuneus
	82	0.029	5.08	4.86	22	−70	8	R	Calcarine
VICT faces: deactivation	14	0.002	5.72	5.42	20	−42	14	R	Posterior cingulate gyrus
	176	0.010	5.36	5.11	58	−52	42	R	Inferior parietal lobule

*Significant results at FWE-corrected p ≤ 0.05 for clusters consisting of ≥6 voxels are presented. Cluster vx, size in voxels; p (FWE), family-wise error-corrected p-value; T, t-value; Z, z-value equivalent; x, y, z, MNI coordinates of the voxel of maximum significance; R or L, right or left hemisphere; ERC FACES, European Refugee Crisis with close-ups of emotional faces; ERC CROWDS, European Refugee Crisis showing an anonymous crowd; TERR, Islamic terrorists in a threatening pose; VICT FACES, victims of natural disasters or accidents with close-ups of emotional faces*.

#### The Main Effect of Xenophobic Attitudes on Brain Activation in Response to All Documentary Photographs

Comparing the fMRI responses of HX and LX subjects to all four types of documentary photographs (ERC CROWDS, ERC FACES, TERR, and VICT FACES), we did not identify any significant differences between both groups (FWE *p* > 0.05).

#### The Main Effect of Xenophobic Attitudes on Brain Activation by Specific Subcategories of Documentary Photographs

**Figure 6:**
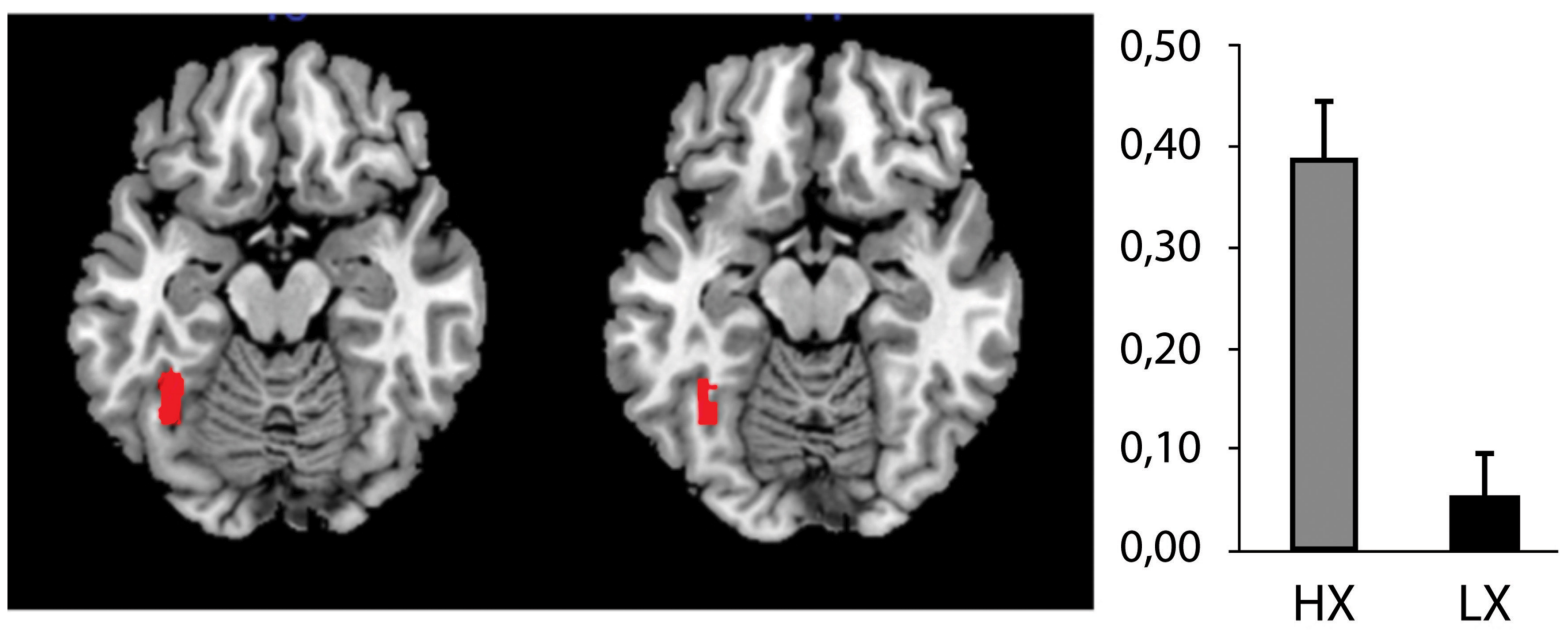
The effect of xenophobic attitudes on brain activation by ERC FACES. The ERC FACES photographs elicited a higher response among the high xenophobia (HX) than among the low xenophobia (LX) subjects (96 voxels, *T* = 5.7, FWE *p* = 0.002, mapped on transversal slices of an anatomical template: MNI *z* = −16, −14). The figure on the right shows the parameter estimates (β values) representing the percentage of blood oxygen level-dependent (BOLD) signal differences between the ERC FACES and the control condition for this fusiform gyrus region.

We identified an effect that was produced by the ERC FACES condition, that is, the image/photograph depicting the refugee crisis with a close-up of emotional faces. Compared to the LX group, the HX group showed increased activation in response to the ERC FACES in the left fusiform gyrus (FWE *p* = 0.002, Figure 6). The LX group (contrasted with the HX) did not exhibit any increased activity.

Interestingly, the increased activation in the fusiform gyrus observed in the HX group was also witnessed in the response to the VICT FACES condition. However, this result was only marginally significant after correcting for multiple testing (FWE *p* = 0.058, cluster of 45 voxels with local maxima *x, y, z* = −38, −48, −16). The LX subjects did not exhibit more activation than HX subjects in any brain region. The xenophobia factor was not found to have any significant effect at the *p* ≤ 0.05 FWE-corrected threshold for any of the other conditions (ERC CROWDS, TERR). The factors of photographic content (four levels: ERC CROWDS, ERC FACES, TERR, and VICT FACES) and group (two levels: HX and LX) showed an interaction in the left fusiform gyrus (*x, y, z* = −38, −52, −16) and two clusters in the cerebellum (*x, y, z* = −36, −60, −42; −26, −74, −36), but these findings did not survive the FWE correction.

#### The Effect of Xenophobic Attitudes on the Functional Connectivity of the Left Fusiform Gyrus During the Presentation of Stimuli Depicting the Refugee Crisis With a Close-Up of Emotional Faces (ERC FACES) Condition

To explain why the HX subjects exerted a higher BOLD response to ERC FACES in the left fusiform gyrus than the LX participants (the main finding of this study), we performed a functional connectivity (FC) analysis of this ROI. This analysis sought to identify the differences between the two groups in the ERC FACES condition-specific regulation of FG. First, we created the mask of the fusiform gyrus activation that was identified by full-factorial SPM contrast for the ERC FACES condition thresholded at FWE *p* ≤ 0.05. Second, we calculated the correlation between the residual BOLD time course from this left FG seed consisting of 96 voxels (local maximum: *x, y, z* = −36, −58, −16) and all the other gray matter voxels for the ERC FACES in both the HX and LX groups (this 2 × 4 ANOVA approach mirrors the main SPM model used for BOLD mapping). For the ERC FACES condition, we found higher FC between the left fusiform gyrus seed and the clusters of the right middle frontal gyrus, the left supplementary motor area, and the right precuneus (*p* ≤ 0.05 FWE corrected) among HX subjects than LX subjects.

**Figure 7:**
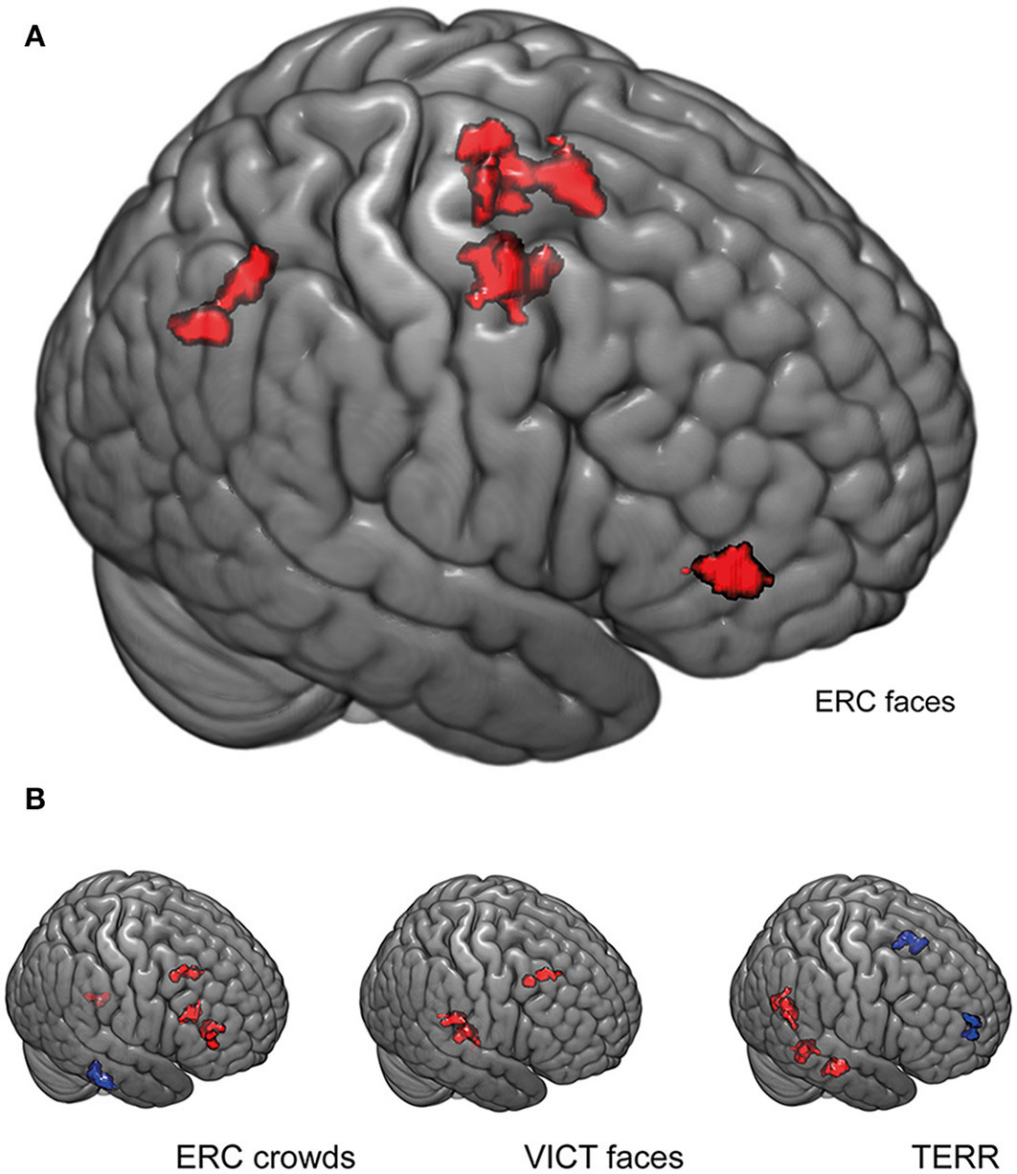
Functional connectivity (FC) analysis of the left fusiform gyrus. **(A)** For the ERC FACES condition, differences were observed between HX and LX subjects in the functional connectivity of the left fusiform gyrus seed (functional cluster of the left FG; FWE *p* ≤ 0.05, 96 voxels, local maxima *x, y, z* = −36, −58, −16). The FC is higher among HX subjects for the right middle frontal gyrus, the left supplementary motor area, and the precuneus. This ERC FACES-specific pattern of the FG FC differed from the patterns observed for the other conditions shown in **(B)**. For each condition the color red indicates increased functional connectivity (with the left FG seed) among HX subjects compared to LX subjects, and the color blue indicates increased FC among LX subjects compared to HX subjects (*p* ≤ 0.05 FWE–corrected). The bar shows the image intensity brightness range from 0 to 0.5. ERC FACES: European refugee crisis with a close-up of emotional faces; ERC crowds: European refugee crisis showing an anonymous crowd; TERR: Islamic terrorists in a threatening pose; VICT FACES: victims of natural disasters or accidents with a close-up of emotional faces. Clusters are rendered on an MNI template using MRIcroGL (https://www.nitrc.org/projects/mricrogl/).

Interestingly this connectivity pattern was condition-specific for the ERC FACES and differed from the patterns observed for the ERC CROWDS (compared to LX subjects, HX subjects showed increased FC in the right inferior, middle, and superior frontal gyri and in the left cerebellum, and decreased FC in the brainstem), the TERR (compared to LX subjects, HX subjects showed increased FC in the right lingual, parahippocampal gyri, and the distinct cluster of the fusiform gyrus, and decreased FC in the left operculum and the precentral gyrus), and the VICT FACES (compared to LX subjects, HX subjects showed increased FC in the right middle frontal and the superior temporal gyrus) (Figure 7, Table 3).

**Table 3:** Functional connectivity analysis of the left fusiform gyrus seed.

**Condition**	***x***	***y***	***z***	**No. voxels**	**Cluster *p*-FWE**	**R or L**	**Region**
ERC FACES	−8	−6	56	122	0.000	L	Supplementary motor area
	28	−2	52	119	0.000	R	Middle frontal gyrus
	4	−68	28	62	0.023	R	Precuneus
ERC CROWDS	42	38	12	87	0.003	R	Inferior frontal gyrus
	12	−44	−58	77	0.007	R	Cerebellum
	28	26	46	72	0.011	R	Middle frontal gyrus
TERR	30	−60	2	133	0.000	R	Lingual gyrus
	32	−52	−20	103	0.001	R	Fusiform gyrus
	−50	−18	16	98	0.002	L	Post-central gyrus
	34	−22	−28	75	0.010	R	Parahippocampal gyrus
VICT FACES	52	−24	10	154	0.000	R	Superior temporal gyrus
	32	14	42	49	0.074	R	Middle frontal gyrus

*Significant results at FWE-corrected p ≤ 0.05. x, y, z, MNI coordinates of the voxel of maximum significance; R or L, right or left hemisphere; ERC FACES, European refugee crisis with a close-up of emotional faces; ERC CROWDS, European refugee crisis showing an anonymous crowd; TERR, Islamic terrorists in a threatening pose; VICT FACES, victims of natural disasters or accidents with a close-up of emotional faces*.

## Discussion

In this study we sought to determine how individual attitudes to the European refugee crisis correspond to selected psychological state and traits measures and how such attitudes affect the neural processing of media images of refugees. The results of psychometric measurements revealed that psychological biases shaped people's attitudes toward immigrants who recently arrived in Europe. Most notably, we found that a negative attitude toward immigrants (high xenophobia) was associated with higher trait aggression and hostility and a significantly higher conscientiousness score, while no group effect was found for empathy or anxiety measures. We also demonstrated that pre-existing attitudes toward refugees affected brain response to media images of refugees. The viewing of images of refugees with emotional faces was associated with enhanced activity in the left fusiform gyrus in the high-xenophobia participants. The low xenophobia group was not associated with any increased activation for any condition. The neuroimaging results do not conform to typical differences between in-group vs. out-group perception.

### The Processing of Affective Faces

Regarding the response to representations of refugees with emotional faces, our aim was to adjudicate between two possibilities—the first being that the low xenophobia group would show patterns of neural response connected with in-group empathy bias and the enhanced processing of faces that is connected to this bias. The alternative scenario predicted there would be an enhanced processing of faces in the high xenophobia group reflecting increased attention and vigilance. Our results clearly support the second alternative. The fact that there were no differences in measures of empathy between the LX and HX groups is consistent with the finding of absence of activation in the brain structures that are typically involved in empathy for the pain of others in the less xenophobic group. Moreover, no amplification of FG activity was registered in the LX group, which could be interpreted as a sign of in-group bias against refugees. Consistent with the second possibility, in the more xenophobic group there was enhanced activation in the FG in response to images of refugees with close-ups of facial expressions. This is consistent with previous research that has produced strong evidence showing that cognitive and perceptual resources are—often without conscious awareness—allocated to processing salient individuals and groups who appear to pose the major functional implications for the perceivers (Maner et al., 2005; Ackerman et al., 2006) and that the faces of out-group members who are perceived negatively elicit increased attention and vigilance. Most of these studies examined the effect of implicit racial bias and observed evidence of attentional prioritization of racial out-groups (Eberhardt et al., 2003; Ito and Urland, 2005; Donders et al., 2008; Dotsch et al., 2008; Trawalter et al., 2008). Also relevant in this context is the finding from the study by Dickter et al. (2015), who found that contact with racial out-group members moderates the allocation of attention to out-group faces, but this social contact must be close and extended in nature. Moreover, several studies that used dot-probe tasks suggest that even when out-groups and in-groups do not differ in terms of the degree of threat that is associated with them, subjects still demonstrate greater attentional allocation to out-group than in-group faces (Al-Janabi et al., 2012; Navarrete et al., 2012; Brosch et al., 2013).

It is already well-established that there is increased reactivity in the fusiform gyrus to a potentially threatening face (Vuilleumier et al., 2003; Petrovic et al., 2008). To date we do not have extensive neuroimaging evidence on the role of the FG in social perception, but it has been established that group membership has a top-down effect on FG activity, albeit with contradictory results. While some studies found enhanced activity in the FG in response to racial or minimal in-group faces compared to out-group faces (Golby et al., 2001; Van Bavel et al., 2011), the opposite pattern—which is evident in our results—is in line with the findings of some other related studies. Using multivoxel pattern analysis, Brosch et al. (2013) found that patterns of response to visual representations of black and white faces were more varied in (White) subjects with higher levels of implicit pro-white bias (Brosch et al., 2013). Molapour et al. (2015) found activity increasing over time in bilateral fusiform gyrus (along with amygdala and right hippocampus) to racial outgroup (Black) as compared to White faces. Interestingly, the fusiform activity in the HX group was also slightly increased in response to the category of images showing emotional suffering in other, non-refugee contexts (VICT FACES).

Our main finding that left fusiform activity corresponds to stronger xenophobic attitudes can be understood in the light of new findings and models concerning social vision (Eberhardt et al., 2004; Adams et al., 2011; Ratner et al., 2014; Freeman and Ambady, 2014; Johnson et al., 2015) and specifically in connection with the dynamic interactive model of social perception that was recently outlined by Stolier and Freeman (2016). Their model posits that visual perception of social categories is the result of lower-level face processing and higher-order social cognition, including stereotypes and attitudes, that mutually constrain and reinforce each other in a feedback loop. Facial cues activate categories and stereotypes that are associated with these cues, and these stereotypes in turn constrain category activation itself. In this way stereotypes and attitudes can impact even initial categorization. While, as noted above, refugees arriving in Europe during the ERC were ethnically, religiously, and culturally heterogeneous, the representations in the media presented salient visual cues (the images are often of darker-pigmented people) and contextual (group) cues that instantly activated both a particular social category (“REFUGEE”) and the observer's attitude toward this category, prompting enhanced processing. Arguably, in more xenophobic subjects, the bidirectional associations between images of refugees and the stereotypes they elicit operate, as Eberhardt et al. (2004) suggest, like visual tuning devices that impact the response during relatively early stages of processing. That the HX group perceives refugees as a high-salience issue is thus likely a factor in biasing attention to their faces and their increased processing in fusiform gyrus, but is not sufficient to register in the measurable activity in affect-related brain areas. This is consistent with the fact that group cues in immigration news may trigger anxiety independently of actual information about the severity of the threat (Brader et al., 2008). Given that most of the stimuli in this category depicted non-white individuals, it is possible that the HX subjects are more vigilant whenever they are confronted with representations of out-group faces, irrespective of the context.

Importantly, while our results indicate increased attention and vigilance toward ERC-related stimuli in the HX group, they do not show the patterns of response that are associated with dehumanized out-group perception, such as reduced MPFC and enhanced amygdala and insula activation (Harris and Fiske, 2009). This may seem at odds with the fact that refugees generally are often the subject of dehumanization (Utych, 2018) and specifically with the results of a recent population survey, which found strong opposition to refugees on all measures and their blatant dehumanization by the Czech population (Bruneau et al., 2017). The most likely explanation is the fact that the negative attitudes toward refugees in our HX group were not as pronounced as they are among the most strongly anti-refugee segments of the general population. This is the trade-off for being able to study a homogenous group of subjects, such as university students enrolled in the same field of study. When compared to a representative sample of the Czech general population, our participants showed a significantly lower level of xenophobia (Eurobarometer, 2015). Only 16.7% of medical students exhibited high xenophobia (FXS score lower than 0.4), compared to 74.7% of a sample of the Czech general population. Also, 46.5% of medical students showed the lowest xenophobia level (FXS score 0.6–1), while this was true of only 5.9% of the general population (Buchtík et al., 2015).

### FG Activity and Personality Traits

This interpretation of increased FG activity in HX subjects is further supported by our finding that the HX subjects scored significantly higher in conscientiousness. As an extensive body of research has shown, out of all personality traits, conscientiousness, along with openness, has the strongest influence on political ideology. Consciousness strongly correlates with conservatism, right-wing authoritarianism, and measures of moral traditionalism and attitudes regarding social, economic, and security issues (Sibley and Duckitt, 2008; Gerber et al., 2010). In a similar vein, a recent study from the UK revealed that reduced subjective and objective cognitive flexibility is associated with more authoritarian, nationalistic, and conservative ideological orientations, which in turn corresponds to support for Brexit and opposition to immigration (Zmigrod et al., 2018). A related body of research in political psychology has demonstrated that physiological responsiveness to threat cues correlates with the degree to which individuals advocate policies that protect the existing social structure from both out-group and internal norm-violators (Oxley et al., 2008). Conservative and right-of-center attitudes are associated with stronger negativity bias; that is, increased attention and greater physiological responsiveness to aversive stimuli (Dodd et al., 2012; Hibbing et al., 2014). These findings thus provide direct support for the tripartite association between more pronounced anti-refugee attitudes, conscientiousness, and enhanced fusiform activity in our HX group.

Admittedly, an alternative explanation for the relationship between an increased measure of conscientiousness and enhanced fusiform activity in HX is theoretically admissible, if less likely. Assuming that greater activation of the FG is associated with time spent fixating on facial expressions (Dalton et al., 2005), it could be argued that the (more conscientious) HX subjects may have simply better fulfilled the instruction to internalize and feel the emotion evoked by the stimuli. HX subjects may thus have been struggling to accomplish the task by fixating on the faces in the close-up images of emotional expressions.

### The Absence of Emotion-Related Processing

As expected, we found right amygdala activity in response to the images of ISIS terrorists in explicitly threatening postures for both groups. However, there was no increased activation in the amygdala or other brain structures associated with anxiety and fear, such as the ventromedial prefrontal cortex, the insula, the hippocampus, and the PAG (Satpute et al., 2012; Rigoli et al., 2016) for two categories of refugee images, that is, crowds and emotional faces. This contrasts with the findings of several previous studies that found amygdala reactivity to faces from racial and dehumanized out-groups, often stereotypically perceived as representing a threat and a danger (Cunningham et al., 2004; Lieberman et al., 2005; Stanley et al., 2008; Amodio and Hamilton, 2012; Chekroud et al., 2014). This absence of threat-related activation corresponds to the results of our psychological assessment, as we found no correlation between negative attitudes and psychological measures of anxiety and fear and no group-specific differences in the measured anxiety level (STAI) or level of fear and phobia (FQ). A simple explanation for this result may be that neither of the two categories of stimuli representing refugees in our experiments were perceived as threatening *per se*, even by those with negative attitudes toward immigrants. As Chekroud et al. (2014) note, it is difficult to make threat-based predictions in experiments, where it is unlikely that the participants will find any stimuli to be directly threatening in that moment. Furthermore, it is likely that in our case it was not possible to elicit a threat-related response sufficient to activate the amygdala because fear and anxiety are “shallowly” encoded, unlike, for example, the threat-related responses of white participants to black faces (e.g., Cunningham et al., 2004; Donders et al., 2008) in a population with a history of long-evolving implicit biases stemming from interracial conflicts. Furthermore, the role of fear in the xenophobic attitudes was previously questioned in general, suggesting that the association of this social phenomena with hatred and hostility is much higher and even proposing more fitting term “xeno-hostility” (Norman, 2004). This assumption is fully supported by our findings showing strong association with hostility and missing link to fear. The presence of increased hostility in our sample with high-xenophobia traits is therefore fully in line with the hostile attitudes toward refugees in public space and media reported in the European Union (cf. Eurobarometer, 2015; Bruneau et al., 2017) present also in studies addressing the direct racial bias (Velasco González et al., 2008). Regarding the absence of the neural response in structures that are typically involved in empathy for the pain of others, a compassion fatigue (Moeller, 1999), arising from an overexposure to negatively valenced media images might also play a role.

### Functional Connectivity

The results of the functional connectivity analysis suggest that the ERC FACE condition evokes in HX subjects increased functional coupling between the identified functional FG cluster and the right prefrontal cortex, the right precuneus, and the left SMA, the area of the brain area responsible for higher cognition (middle frontal gyrus). Since we cannot determine the directionality of this coupling, its interpretation must remain speculative. One possibility is that it may indicate top-down down-regulation of stereotypical, pre-potent response in the FG by the right prefrontal cortex. The increased connectivity between the FG and the SMA in HX subjects might then signal preparation for movement activity in response to facial-salient stimuli identified by the FG (Nguyen et al., 2014).

### Limitations

Some limitations to our study should be noted. The first limitation pertains to the age difference observed between the LX and HX groups. This difference was the result of the distinct maximal value of the age variable in the individual groups. However, both groups were recruited from a single sample of medical students, and the observed age difference was also very small (2 years). Therefore, we argue that this difference should not affect our reported findings. The second limitation is the sample size, which could be a reason for some of the missing group differences in brain activity. The group sizes were comparable or larger than in other key studies in this subject area (Bruneau and Saxe, 2010; Mathur et al., 2010; Bruneau et al., 2012; Gutsell and Inzlicht, 2012) that reported fMRI-based differences in processing in-group/out-group related stimuli. The current study samples were also selected from an otherwise quite homogenous population (at least in age and education), which should lead to a higher signal-to-noise ratio due to decreased spurious interindividual variability.

Also, while the student sample in general tends toward lower xenophobia, this is a shift rather than a narrowing of the range of xenophobia measures, so in principle this tendency does not decrease the power of the validity of the findings *per se*. To optimize power of the study, we have used an extreme groups design. In particular, from a sample of 217 subjects, we have selected those at the extreme ends of the xenophobic scale (in particular 19 and 19 from each extreme). Comparison of samples of this size by a independent samples *t*-test provides 80% power to detect an effect of size *d* = 0.95 (Cohen's d), and power 67% to detect an effect of size *d* = 0.8. While such effect sizes may be classified as “large,” note that we are indeed expecting large effect sizes in the group comparison, as only the extremal groups are being compared.

Of course, the general separation of the high xenophobia and low xenophobia group based on the Fear-based Xenophobia Scale might be suboptimal. While currently it is probably the only measure that is used internationally and was developed and validated for psychometric testing (Canetti-Nisim and Pedhazur, 2003; Van der Veer et al., 2011—Czech translation Tabery et al., 2015) and that has also been shown to be reliable and valid for use on the Czech population (Kunc, 2016), it is of course far from perfect and may to some extent suffer by various problems related to self-reporting in general. However, the extreme groups identified based on Fear-based Xenophobia Scale clearly showed significant differences in multiple psychological and neuroimaging variables studied here. Future studies using the full range of subjects, or alternative criteria, or further improved Fear-based Xenophobia Scale may be warranted.

It also needs to be acknowledged that our stimuli consisted of isolated images, whereas media (TV news, web or printed sources) almost invariably present composite image-texts, in which visual representations are accompanied by headlines, captions, comments, or spoken narratives. The design of our study did not allow for analyzing the effect of such multimodal interaction between visual and textual components.

Finally, we are fully aware that the neuropsychological approach (MRI imaging and psychometric methods) applied allows only for psychological interpretation of the obtained findings, while the broader sociological perspective is missing and should be considered in future studies.

## Conclusion and Implications

In this study, we found differences both in some psychological traits and in brain activity between HX and LX subjects when viewing images of ERC refugees. However, the results do not conform to the typical patterns of distinction between in-group vs. out-group perception, and it is worth briefly spelling out the possible implications of this. On the one hand, the response in the low xenophobia group was not consistent with the neural signature of in-group bias. At the same, while we found increased attention and vigilance toward ERC-related stimuli in the HX group, we did not find patterns of response associated with dehumanized perception of an out-group. In a broader perspective, our results point to the danger of the self-perpetuating vicious circle that underlies the way in which media images of refugees are perceived: attitudes and stereotypes that are preformed (mostly from previous exposure to media representations) elicit a rapid social categorization that confirms and reinforces the existing attitude, leaving little room or need for a reflective processing of media image-texts and their often ambiguous and complex messages. This would support previous evidence that people selectively interpret media news in a way that affirms their pre-existing opinions and attitudes (Vallone et al., 1985; Quillian, 2006). At the same time, this interpretation need not be incompatible with the evidence for media-induced xenophobia (and Islamophobia specifically) (Das et al., 2009; Shaver et al., 2017). Presumably, the increased exposure to negative news and social media messages about immigration, refugees, and related topics accruing over time, augmented by communication with members of peer groups, forms a cognitive bias, which then further drives the response to news featuring refugees. Future research, combining approaches drawn from sociology, media studies, psychology, and cognitive neuroscience, thus needs to focus on investigating the micro-dynamics of the relationship between exposure to media, attitude formation, and responses to media image-texts focused on refugees.

## Data Availability Statement

The datasets generated for this study are available on request to the corresponding author.

## Ethics Statement

The studies involving human participants were reviewed and approved by Ethical Committee of the National Institute of Mental Health. The participants provided their written informed consent to participate in this study.

## Author Contributions

LK and JHo: conceptualization, funding acquisition, and supervision. PA, DGry, JT, MB, and AA: data curation. PA, DGre, IF, TN, PK, MB, AA, and JHl: formal analysis. LK, IF, PA, MB, and DGry: investigation. LK, JHo, IF, TN, FŠ, JT, JHl, and MB: methodology. DGry: project administration. DGre, IF, and PA: visualization. LK, JHo, and IF: writing-original draft preparation. LK, IF, PA, MB, DGre, DGry, PK, TN, FŠ, JT, AA, JHo, and JHl: writing-review and editing. All authors contributed to the article and approved the submitted version.

## Conflict of Interest

The authors declare that the research was conducted in the absence of any commercial or financial relationships that could be construed as a potential conflict of interest.
